# Prognostic interaction between clinical stage and left atrial remodeling in dogs with myxomatous mitral valve disease

**DOI:** 10.1080/01652176.2026.2668790

**Published:** 2026-05-06

**Authors:** Sin-Wook Park, Keon Kim, Kyung-Ho You, Woong-Bin Ro, Chang-Min Lee

**Affiliations:** a Department of Veterinary Internal Medicine, College of Veterinary Medicine and BK21 FOUR program, Chonnam National University, Gwangju, Republic of Korea; b SKY Animal Medical Center, Gwangju, Republic of Korea; c Department of Veterinary Internal Medicine, College of Veterinary Medicine, Jeju National University, Jeju, Republic of Korea; d The Care animal medical center, Guri, Gyeonggi-do, Korea

**Keywords:** Canine, cardiogenic pulmonary edema, congestive heart failure, left atrium Echocardiography, prognosis, myxomatous mitral valve disease, ACVIM staging, left atrium to aortic root ratio

## Abstract

Myxomatous mitral valve disease (MMVD) is leading cause of acquired heart failure in small-breed dogs. Although the American College of Veterinary Internal Medicine (ACVIM) staging is widely used in practice, how each stage and the degree of cardiac remodeling affect survival has not been clearly defined. We aimed to determine whether severe remodeling in dogs with preclinical (stage B2) disease is associated with survival differences compared with dogs with congestive heart failure (CHF; stage C) and less severe remodeling. This retrospective study analyzed 270 dogs with MMVD, stratified by the left atrium-to-aortic root ratio as having mild (<1.9), moderate (1.9–2.2), and severe (≥2.2) left atrial enlargement. Survival for dogs with stage B2 disease with severe LA enlargement did not differ significantly from dogs with stage C disease with mild-to-moderate enlargement. However, within the subgroup with severe LA enlargement, dogs that developed CHF had significantly shorter survival than those that remained in stage B2 (360 vs. 654 days; *P* = 0.032). Severe LA enlargement in dogs with preclinical disease contributes to a poor prognosis comparable to that of CHF with mild-to-moderate enlargement, indicating that stage alone may be insufficient for determining prognosis and that LA remodeling should also be considered.

## Introduction

Myxomatous mitral valve disease (MMVD) is the most common cardiovascular disorder in small-breed dogs, accounting for approximately 70% of canine cardiac cases (Kim et al. 2017). The progressive degeneration of the mitral valve leads to volume overload and subsequent cardiac remodelling, eventually resulting in congestive heart failure (CHF) (Reynolds et al. 2012; Kim et al. 2017). Dogs with CHF typically present with respiratory distress, exercise intolerance, or collapse, and once CHF develops, the prognosis is poor, with reported median survival times (MSTs) ranging from 136 to 390 days (Borgarelli and Haggstrom 2010; Nakamura et al. 2014; Kim et al. 2017; Park et al. 2025).

The American College of Veterinary Internal Medicine (ACVIM) consensus statement classifies MMVD into stages based on clinical severity (Keene et al. 2019). Dogs with stage B2 disease exhibit radiographic or echocardiographic evidence of cardiomegaly without clinical signs of CHF, whereas stage C includes those with current or previous episodes of CHF requiring lifelong treatment (Häggström et al. 2008). This staging system provides a practical framework for diagnosis and therapeutic decision-making; however, it primarily focuses on clinical management rather than prognostic stratification (Kim et al. 2017; Baron Toaldo et al. 2018).

Echocardiographic indices of cardiac remodelling, particularly the left atrial-to-aortic root ratio (LA/Ao), are key parameters in MMVD evaluation and have been associated with survival (Nakamura et al. 2014; Baron Toaldo et al. 2018; Park et al. 2025). To further refine prognostic assessment, composite echocardiographic indices incorporating parameters such as LA/Ao have been proposed, including the Mitral INsufficiency Echocardiographic (MINE) score. However, this index was derived solely from imaging variables without considering clinical stage or symptomatic status (Vezzosi et al. 2021, 2025). While the onset of CHF is a major determinant of prognosis, the extent of structural remodelling also appears to substantially influence outcome (Kim et al. 2017; Baron Toaldo et al. 2018; Keene et al. 2019). Although survival differences across ACVIM stages have been reported, most studies evaluate stages as discrete categories or as independent risk factors in multivariable models (Vezzosi et al. 2021; Grosso et al. 2023; Svensson et al. 2024). Consequently, the prognostic implications of severe left atrial (LA) remodelling in stage B2 dogs relative to stage C dogs with less marked remodelling remain poorly characterised.

Therefore, this study aimed to evaluate the prognostic relevance of LA remodelling severity in dogs with MMVD in relation to ACVIM clinical stage. Specifically, we investigated whether dogs with stage B2 and severe LA remodelling have survival outcomes comparable to those of dogs with stage C and less marked LA remodelling.

## Materials and methods

### Animals

Medical records from The Care Animal Medical Centre collected between October 2021 and December 2024 were retrospectively reviewed. During this interval, 738 dogs underwent echocardiographic examination for evaluation of suspected or known cardiac disease. When MMVD was identified, dogs were staged according to the ACVIM classification (B1–D) based on echocardiographic and clinical findings (Keene et al. 2019).

Dogs were eligible for inclusion if they were ≥ 6 years old, ≤ 15 kg, had MMVD staged as ACVIM B1, B2, or C, and had no prior diuretic administration before the presentation. The exclusion criteria were the presence of congenital cardiac abnormalities or other acquired cardiac diseases (e.g. bacterial endocarditis, dilated cardiomyopathy, pulmonary stenosis).

Dogs classified as having stage B2 disease were required to meet the ACVIM consensus criteria for preclinical MMVD, including a normalised left ventricular internal diameter in diastole (LVIDdN) ≥ 1.7 and LA enlargement (LA/Ao ≥ 1.6). The baseline for longitudinal analyses was defined as follows: for dogs previously determined to have stage B1 disease at our hospital, the date when progression to stage B2 disease was first confirmed; for dogs initially diagnosed with stage B2 disease at our hospital, the date of the initial stage B2 diagnosis; and for dogs previously diagnosed with stage B2 disease at another facility, the date of initial confirmation at our hospital. Dogs that later developed CHF were not double-counted and were analysed according to their baseline stage (B2). This approach was used to ensure a consistent and verifiable baseline time point for survival-time calculation and to anchor longitudinal echocardiographic variables to the first standardised assessment at our hospital.

Dogs classified with stage C disease were defined as those with MMVD and a first-onset episode of CHF, diagnosed based on tachypnea/dyspnoea or respiratory distress together with thoracic radiographic evidence of pulmonary oedema. Dogs that had previously been diagnosed as having stage B1 disease at our hospital but first presented with CHF were also classified as having stage C disease.

Dogs were further stratified according to the LA/Ao into three remodelling categories: mild (<1.9), moderate (1.9–2.2), and severe (≥2.2). These thresholds were based on a previously proposed ordinal classification of LA enlargement using LA/Ao, and the upper cut-point was slightly adapted to maintain adequate subgroup sizes for subsequent survival analyses (Rishniw 2023). In addition, 50 dogs classified as having stage B1 disease according to the ACVIM criteria were included for baseline echocardiographic comparison. These dogs were not incorporated into survival analyses but were analysed descriptively to provide reference values for structural and functional indices.

### Echocardiographic data

**Figure 1.**
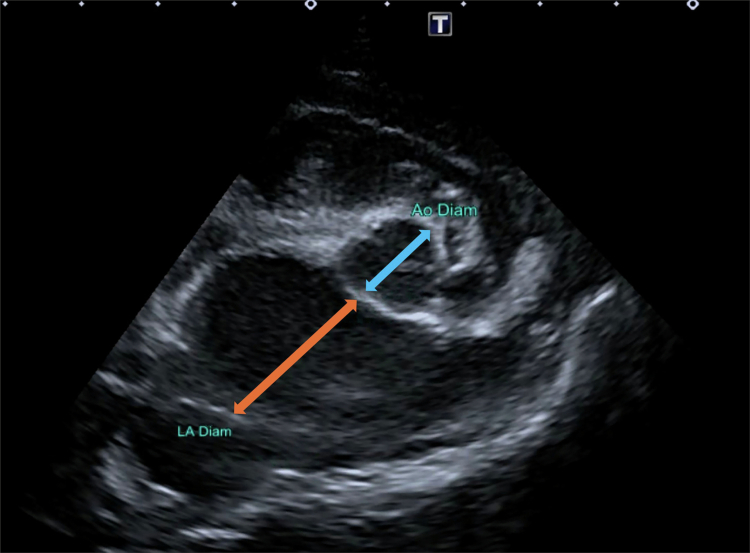
Measurement of the left atrial-to-aortic root ratio (LA/Ao) from the right parasternal short-axis two-dimensional view at the level of the aortic valve. The blue and orange arrows indicate the aortic and left atrial diameters, respectively.

All echocardiographic examinations were performed by two board-certified veterinary radiologists and one board-certified veterinary internist and subsequently reviewed by another board-certified veterinary internist. The value recorded for each measurement consisted of the average of three cardiac cycles. The LA/Ao was obtained from the right parasternal short-axis two-dimensional (2D) view as previously described (Hansson et al. 2002), as illustrated in Figure 1, and the left ventricular internal diameter at end-diastole (LVIDd) and left ventricular internal diameter at end-systole (LVIDs) measured on the M-mode echocardiogram were obtained from the right parasternal short-axis view. M-mode values were used to derive the fractional shortening percentage (FS%). Normalised dimensions were calculated according to the following formula: LVIDdN = LVIDd(cm)/(BW (kg))^0.294^ (Boswood et al. 2018).

From a left apical 4-chamber view, the peak velocity of the early diastolic transmitral flow (E) was obtained. The early diastolic mitral annular velocity (E′) was measured by tissue Doppler imaging at the septal and/or lateral annulus, and the ratio of E to E′ (E/E′) was subsequently calculated. The maximal flow velocity of the tricuspid regurgitation (TR) jet from the following three different views was assessed: left parasternal (LPS) apical 4-chamber view, LPS long-axis view of the right auricle, and LPS cranial transverse view of the tricuspid valve. This measurement was then applied to estimate the systolic pressure gradient across the tricuspid valve, representing the systolic pulmonary artery pressure.

A diagnosis of PH was made according to the ACVIM consensus statement, which classifies the likelihood of PH as low, intermediate, or high based on the peak TR velocity in combination with the number of supportive echocardiographic signs observed at different anatomic sites (Reinero et al. 2020). In dogs with stage C disease presenting with acute pulmonary oedema, the TR velocity was re-evaluated after stabilisation with diuretic therapy, as this assessment was considered to be more reliable once pulmonary congestion had improved.

### Statistical analysis

All statistical data were analysed using commercial software (IBM SPSS Statistics, version 27, IBM Co., United States). Data were tested for normality using the Shapiro-Wilk test. Descriptive statistics are reported as the mean ± standard deviation (SD) for normally distributed continuous variables and as the median (interquartile range, IQR) for non-normally distributed continuous variables. For comparisons between two groups, the Chi-square test was used for categorical variables, and an independent samples t-test or the Mann–Whitney U-test was used for continuous variables. In addition, for comparisons among the three groups, the Chi-square test was used for categorical variables, and a one-way ANOVA or Kruskal–Wallis test was used for continuous variables depending on the data distribution. When significant differences were detected, post hoc pairwise comparisons were performed using Bonferroni or Dunn’s correction as appropriate.

Dogs were censored at the time of last follow-up if they were alive at the study conclusion, lost to follow-up, or died due to non-cardiac causes. The primary endpoint for survival analysis was cardiac-related death. Cardiac-related death was defined as death or euthanasia preceded by refractory respiratory distress consistent with CHF or cardiogenic shock/low-output signs attributable to MMVD. Deaths with an undetermined cause were classified as non-cardiac/undetermined and were treated as non-cardiac for censoring purposes. Accordingly, dogs were censored at the date of last confirmed contact if they were alive at study conclusion or lost to follow-up, and at the date of death if they died due to non-cardiac/undetermined causes. Univariable Cox proportional hazards analysis (age, sex, body weight, creatinine, LVIDdN, FS, E peak, E/E’ ratio, MMVD stage [B2 vs C], LA/Ao group [LA/Ao < 1.9, 1.9–2.2, ≥ 2.2], and probability of PH [low, intermediate, high]) was performed to identify potential predictors of cardiac-related death by calculating hazard ratios (HRs) with 95% confidence intervals (CIs). The significant factors (LVIDdN, E/E’ ratio, MMVD stage, and LA/Ao group) from the univariable analysis (*P* < 0.2) were included in the multivariable Cox proportional hazards analysis. Kaplan–Meier survival curves with log-rank tests were employed to compare the MSTs between groups. Survival time was calculated from the date of each dog’s first diagnosis of stage B2 or stage C disease to the date of cardiac-related death or censoring. Statistical significance was set at *P* < 0.05.

## Results

### Study population

During the study period, a total of 738 dogs that underwent echocardiographic examination were evaluated. Of these, 361 were classified as having stage B1 disease, 137 as having stage B2 disease, 133 as having stage C disease, and 9 as having stage D disease. Other cardiac diagnoses included heartworm disease (*n* = 15), patent ductus arteriosus (*n* = 10), dilated cardiomyopathy (*n* = 3), hypertrophic cardiomyopathy (*n* = 3), cardiac tumour (*n* = 3), ventricular septal defect (*n* = 1), peritoneopericardial diaphragmatic hernia (*n* = 1), and tetralogy of Fallot (*n* = 1). In addition, 18 dogs had PH without evidence of left-sided cardiac abnormalities, and 43 dogs had no remarkable echocardiographic findings. Based on the predefined inclusion criteria, 128 dogs classified as having stage B2 disease and 92 dogs classified as having stage C disease were included in the final analysis. In addition, 50 dogs with stage B1 disease were included for baseline comparisons but were not entered into the survival analyses.

The baseline characteristics of dogs with stage B1, B2, and C disease are summarised in Table 1. A total of 270 client-owned dogs were enroled in the study. The most frequently represented breeds were Maltese (*n* = 108), mixed breed (*n* = 43), Pomeranian (*n* = 35), Poodle (*n* = 31; no Standard Poodles), Shih Tzu (*n* = 20), and Chihuahua (*n* = 11). Several other breeds were represented in smaller numbers, including Yorkshire Terrier (*n* = 8), Spitz (*n* = 5), Cocker Spaniel (*n* = 3), Schnauzer (*n* = 2), Dachshund (*n* = 2), West Highland Terrier (*n* = 1), and Pekingese (*n* = 1). Overall, 152 dogs were male, including 135 that were neutered, and 118 were female, including 93 that were spayed. In the stage B2 group (*n* = 128), 73 dogs had an LA/Ao < 1.9, 32 had an LA/Ao between 1.9 and 2.2, and 23 had an LA/Ao ≥ 2.2. In the stage C group (*n* = 92), 25 dogs had an LA/Ao < 1.9, 32 had an LA/Ao between 1.9 and 2.2, and 35 had an LA/Ao ≥ 2.2.

**Table 1.** Descriptive statistics for all 270 dogs with ACVIM stages B1, B2, and C MMVD.

Variable	Stage B1 (*n* = 50)	Stage B2 (*n* = 128)	Stage C (*n* = 92)	*P*-value
Age (years)	10.5 ± 2.5	11.7 ± 2.3	11.3 ± 2.2	0.056
Sex (male/female)	(28/22) (56%/44%)	76/52 (59%/41%)	48/44 (52%/48%)	0.568
Body weight (kg)	4.4 (3.6–5.7)	4.2 (3.2–5.4)	3.9 (3.0–4.8)	0.104
Heart rate (beats per minute)	148 (126–158)	156 (130–180)	150 (126–168)	0.425
Systolic arterial blood pressure (mmHg)	144 (126–158)	133 (123–152)	132 (118–148)	0.212
Creatinine (mg/dL)	0.9 (0.6–1.1)	0.9 (0.7–1.2)	0.9 (0.7–1)	0.476
LVIDdN	1.38 (1.32–1.57)^a^	1.87 (1.77–2.04)^b^	1.94 (1.75–2.17)^c^	<0.001
FS (%)	53 ± 11	55 ± 9	57 ± 11	0.265
LA/Ao	1.30(1.16–1.44)^a^	1.85 (1.70–2.08)^b^	2.07 (1.83–2.36)^c^	<0.001
E peak (cm/s)	74 ± 16^a^	126 ± 29^b^	143 ± 32^c^	<0.001
E/E’ ratio	10.4 (8.6–12.4)^a^	14.2 (11.6–17.1)^b^	19.3 (15.9–23.8)^c^	<0.001
Probability of PH (low/intermediate/high) (%)	41/6/3 (82%/12%/6%)	93/20/15 (72%/16%/12%)	65/15/12 (71%/16%/13%)	0.635

^a,b,c^ Means within a row with different superscripts differ significantly (P<0.05). Continuous variables were compared among groups using one-way ANOVA (normally distributed data) with Tukey’s post-hoc test, or the Kruskal–Wallis test (non-normally distributed data) with Dunn’s post-hoc test with Bonferroni adjustment, as appropriate. Categorical variables were compared using the chi-square test or Fisher’s exact test, as appropriate. E peak, peak velocity of E wave of transmitral flow; E/E’ ratio, ratio of transmitral E wave velocity to early diastolic mitral annular velocity measured by tissue Doppler imaging; FS, fractional shortening; LA/Ao, left atrium-to-aortic root ratio; LVIDdN, normalised left ventricular end-diastolic internal diameter indexed; PH, pulmonary hypertension.

Across the three stages, echocardiographic variables including the LVIDdN, LA/Ao, E peak, and E/E′ ratio increased progressively from stage B1 to B2 and from stage B2 to C (*P* < 0.05). In contrast, age, sex, body weight, heart rate, systolic arterial blood pressure, plasma creatinine concentration, FS, and the probability of PH did not differ significantly among the three stages (Table 1).

### Treatments

Discharge medications were summarised only for dogs included in the survival analyses that survived to discharge. Among dogs with stage B2 disease, 106 of 128 dogs received cardiac medications at discharge, all of whom were administered pimobendan. Additional drugs included enalapril (15/106), spironolactone (2/106), and sildenafil (12/106). Among the 92 dogs with stage C disease included in the final analysis, 87 received cardiac medications at discharge (all pimobendan); the remaining 5 dogs did not survive to discharge and therefore had no discharge prescriptions. Many additionally received loop diuretics such as furosemide (45/87) or torsemide (42/87), while others were treated with enalapril (15/87), spironolactone (75/87), sildenafil (8/87), and isosorbide dinitrate (26/87). The median (range) doses of each drug administered at discharge are as follows: pimobendan, 0.3 mg/kg BID (0.25–0.35) in stage B2 and 0.3 mg/kg BID (0.3–0.4) in stage C; enalapril, 0.5 mg/kg BID (0.5–1.0); spironolactone, 1.0 mg/kg BID; sildenafil, 1.0 mg/kg BID (0.5–1.0) in stage B2 and 1.25 mg/kg BID (1.0–1.5) in stage C; torsemide, 0.2 mg/kg BID (0.1–0.3); furosemide, 1.8 mg/kg BID (1.0–2.5); and isosorbide dinitrate, 0.5 mg/kg BID (0.5–1.0).

### Prognostic factors

Of the 220 dogs with stage B2 or C MMVD, 65 reached the cardiac-related endpoint, including 5 dogs that underwent euthanasia. Additionally, 22 dogs died of non-cardiac causes, 47 were alive at the end of the study, and 86 were lost to follow-up. The results of the univariable Cox proportional hazards analysis in dogs with stage B2 and C disease are shown in Table 2. Variables with a *P*-value < 0.2 in the univariate analysis, namely, the LVIDdN, E/E’ ratio, MMVD stage, and LA/Ao group, were entered into the multivariable Cox regression model. In the final model, both the MMVD stage and LA/Ao group remained independently associated with survival. Dogs classified as having stage C disease had a markedly increased hazard of death compared with those with stage B2 disease (HR = 2.297, 95% CI 1.319–4.002, *P* = 0.003). Dogs with an LA/Ao ≥ 2.2 had a significantly higher hazard of death compared with those with an LA/Ao < 1.9 (HR = 2.334, 95% CI 1.219–4.468, *P* = 0.011), whereas moderate enlargement (LA/Ao 1.9–2.2) was not significantly different (HR = 1.466, 95% CI 0.713–3.014, *P* = 0.298). Neither the LVIDdN (*P* = 0.578) nor the E/E′ ratio (*P* = 0.363) remained significant predictors in the final multivariable model.

**Table 2.** Univariate Cox proportional hazards regression associated with outcome in dogs with stage B2 and C MMVD (*n* = 220).

Variable	Hazard ratio	95.0% confidence interval		*P*-value
Age (years)	1.031	0.924	1.152	0.584
Sex	1.088	0.842	1.405	0.520
Body weight (kg)	0.991	0.880	1.117	0.888
Creatinine (mg/dL)	1.298	0.863	1.955	0.211
LVIDdN	3.535	1.231	10.150	0.019
FS (%)	1.019	0.989	1.050	0.211
E peak (cm/s)	1.003	0.995	1.011	0.430
E/E’ ratio	1.046	1.011	1.081	0.009
MMVD stage (B2 vs C)	2.625	1.553	4.435	<0.001
**LA/Ao group**				0.004
LA/Ao < 1.9 (ref)	1.0	–	–	–
LA/Ao 1.9–2.2	1.713	0.882	3.328	0.112
LA/Ao ≥ 2.2	2.754	1.491	5.084	0.001
**Probability of PH**				0.689
Low (ref)	1.0	–	–	–
Intermediate	1.315	0.660	2.618	0.436
High	1.251	0.496	3.151	0.635

Sex, MMVD stage, LA/Ao group, and PH group were entered as categorical variables. E peak, peak velocity of E wave of transmitral flow; E/E′ ratio, ratio of transmitral E wave velocity to early diastolic mitral annular velocity measured by tissue Doppler imaging; FS, fractional shortening; LA/Ao, left atrium-to-aortic root ratio; LVIDdN, normalised left ventricular end-diastolic internal diameter indexed to body weight; MMVD, myxomatous mitral valve disease; PH, pulmonary hypertension.

### Survival analysis

**Figure 2.**
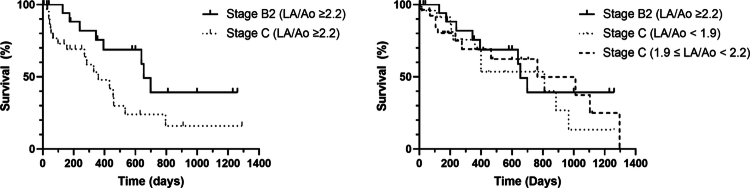
Kaplan–Meier survival curves comparing survival times according to the ACVIM stage and degree of LA remodeling in dogs with MMVD. (A) Comparison between dogs with stage B2 and stage C disease with severe LA enlargement (LA/Ao ≥ 2.2). Dogs with stage C disease showed significantly shorter survival than those with stage B2 disease (median survival time, 360 vs 654 days; *P* = 0.032). (B) Comparison of dogs with stage B2 disease with severe LA enlargement (LA/Ao ≥2.2) and those with stage C disease stratified by LA/Ao category (<1.9, 1.9–2.2, and ≥2.2). Dogs with stage B2 disease with marked remodeling exhibited survival comparable to that of dogs with stage C disease with mild or moderate LA enlargement (*P* = 0.804). Survival distributions were estimated using the Kaplan–Meier method and compared with the log-rank test. The endpoint was cardiac-related death; tick marks indicate censored observations (alive at last follow-up, lost to follow-up, or non-cardiac/undetermined death).

The MST in dogs with stage B2 and C disease was 795 days (95% CI: 564–1,025). Dogs with stage B2 MMVD had a significantly longer MST (1500 days; not reached) than dogs with stage C MMVD, whose MST was 459 days (95% CI: 305–613 *P* < 0.001). In dogs with stage B2 disease, those with severe enlargement (LA/Ao ≥ 2.2) survived a median of 654 days (95% CI: 568–740), which was significantly shorter than those with mild enlargement (LA/Ao < 1.9; 1,500 days; not reached; *P* = 0.042). However, there was no significant difference in overall survival between dogs with severe (654 days, 95% CI: 568–740) and moderate (1140 days, 95% CI: 340–1940) LA enlargement (log-rank test, *P* = 0.984). In dogs with stage C disease, those with severe LA enlargement (LA/Ao ≥ 2.2) had the shortest MST (360 days, 95% CI: 175–545) compared with dogs with mild (810 days, 95% CI: 348–1272) or moderate (765 days, 95% CI: 149–1381) LA enlargement. However, these differences did not reach statistical significance (log-rank test, *P* = 0.183). When directly comparing dogs with stage B2 disease with severe LA enlargement (≥2.2) to those with stage C disease with mild (<1.9) or moderate (1.9–2.2) LA enlargement, no significant difference in survival was observed (654 vs 810 and 765 days; *P* = 0.804). However, within the subgroup of dogs with severe LA enlargement (LA/Ao ≥ 2.2), those with stage C disease had significantly shorter survival (360 vs 654 days; *P* = 0.032) (Figure 2).

## Discussion

This retrospective study investigated the prognostic interaction between clinical stage and the degree of LA remodelling in small-breed dogs with MMVD. Although both the ACVIM stage and LA/Ao are recognised prognostic markers, few studies have examined how structural remodelling across stages influences survival (Nakamura et al. 2014; Baron Toaldo et al. 2018; Keene et al. 2019). Our findings demonstrated that structural remodelling of the left atrium can transcend clinical staging as a determinant of outcome. While stage C remained the strongest predictor of mortality, severe LA enlargement (LA/Ao ≥ 2.2) was independently associated with shorter survival even in dogs with preclinical stage B2 disease. Notably, dogs with stage B2 disease with severe remodelling exhibited a prognosis comparable to that of dogs with stage C disease with only mild or moderate LA enlargement, suggesting that advanced structural disease may offset the prognostic distinction between compensated and decompensated stages.

The clinical stage of MMVD has traditionally been regarded as the principal determinant of prognosis, with stage B2 representing a compensated phase and stage C indicating decompensated disease (Keene et al. 2019; Klein et al. 2021; Pascon et al. 2021). However, the present findings indicate that once severe LA remodelling has occurred, the presence or absence of overt CHF may no longer accurately reflect disease severity. In such cases, assessment of LA remodelling in conjunction with clinical stage may provide a more comprehensive understanding of disease severity and long-term prognosis. This underscores the importance of echocardiographic evaluation of structural progression for risk stratification, even among patients with preclinical disease.

Despite comparable degrees of structural remodelling, dogs with stage C disease with severe LA enlargement exhibited significantly shorter survival than those with stage B2 disease. This finding indicates that the onset of CHF represents a clear clinical classification change, defined by the development of pulmonary oedema and associated clinical signs, while also marking a critical transition in clinical stability. Once decompensation occurs, long-term pharmacologic management and the use of multiple cardiac medications can introduce additional challenges. Chronic diuretic therapy, in particular, may lead to electrolyte disturbances, dehydration, or renal impairment, which can further compromise overall stability and treatment response (Borgarelli and Haggstrom 2010; Ljungvall et al. 2011). These treatment-related factors likely contribute to the marked reduction in survival observed after the onset of CHF, even in dogs with similar degrees of structural disease (Borgarelli et al. 2008). Consequently, once clinical decompensation develops, the prognosis is influenced not only by the severity of cardiac remodelling but also by systemic effects associated with chronic medical therapy.

Within the stage C population, the degree of LA enlargement was not significantly associated with survival, supporting the view that outcomes in decompensated MMVD are influenced by multiple interacting factors rather than structural severity alone. Once CHF develops, persistent neurohormonal activation, elevated afterload, and impaired renal perfusion collectively exacerbate disease progression (Oyama 2009; Borgarelli and Haggstrom 2010). The chronic use of loop diuretics for volume control may also predispose affected dogs to electrolyte disturbances or cardiorenal syndrome, further contributing to shortened survival times (Pouchelon et al. 2015; Orvalho and Cowgill 2017; Ronco et al. 2018; Park et al. 2026). Moreover, because most dogs with stage C disease already demonstrate marked LA and ventricular remodelling, the narrower distribution of the LA/Ao likely reduces the prognostic discriminative power of this marker. Consequently, the prognosis in dogs with stage C disease may be more closely associated with ongoing clinical status including treatment response, owner compliance, and recurrence of CHF than with static echocardiographic measurements (Häggström et al. 2008; Beaumier et al. 2018).

The findings of this study have important clinical implications for the management and risk stratification of dogs with MMVD. Dogs with ACVIM stage B2 disease with marked LA enlargement should not be regarded as a homogeneous preclinical population but rather as a subgroup with limited potential for structural recovery and a prognosis approaching that of early-stage C disease. These results emphasise the importance of evaluating LA remodelling within stage B2 to identify dogs at higher risk of decompensation and to intensify clinical observation and follow-up in these patients. Incorporating LA/Ao-based risk assessment into routine evaluation may therefore help veterinarians allocate greater attention to monitoring and early recognition of clinical deterioration in dogs with preclinical MMVD.

In this study, the prevalence of PH (26%) was comparable to previously reported values in dogs with MMVD, which ranged from 17% to 39% depending on diagnostic criteria and disease stage (Borgarelli et al. 2015; Reinero et al. 2020; Park et al. 2025). PH in MMVD typically develops as a secondary consequence of chronic elevation in LA and pulmonary venous pressures, leading to pulmonary vascular remodelling over time (Reinero et al. 2020). The similar prevalence observed between stages B and C in our cohort suggests that in many dogs with newly decompensated CHF, the LA pressure may not have been chronically elevated long enough to induce significant pulmonary vascular changes. Moreover, PH was not identified as an independent prognostic factor, which may be attributable to the fact that its assessment was performed at a single timepoint at initial diagnosis. Therefore, the potential progression of PH secondary to chronic increases in LA pressure could not be fully reflected. Nevertheless, PH remains an important indicator of advanced disease, and its prognostic relevance might be better demonstrated in populations with prolonged or recurrent elevations in LA and pulmonary venous pressures (Borgarelli et al. 2015; Reinero et al. 2020).

This study has several limitations that should be acknowledged. First, its retrospective design inherently introduces selection and information bias, as treatment protocols, medication dosages, and follow-up intervals were not standardised among cases. Although this heterogeneity limited adjustment for treatment effects in the multivariable model, it may also reflect real-world clinical management of dogs with MMVD, thereby enhancing the practical relevance of the findings. In addition, survival data were incomplete for some dogs because of loss to follow-up, and cardiac-related deaths could not be confirmed in those cases, which may have affected the accuracy of the survival analysis. Censoring was handled according to standard survival analysis methodology, and no evidence suggested systematic bias in follow-up patterns across the study groups. Second, although echocardiographic measurements were performed by experienced clinicians, interobserver variability cannot be completely excluded, which may have affected certain quantitative parameters such as the LA/Ao or LVIDdN (Hsue and Visser 2020; Levicar et al. 2022). Third, the timing of disease classification may have introduced bias, as dogs that progressed from stage B1 to B2 or from B2 to C were analysed based on the echocardiographic data obtained at the first diagnosis of stage B2 disease. Finally, because this was a single-centre study conducted at a hospital, the findings may not be fully generalisable to the broader population of dogs with MMVD seen in primary care settings. Despite these limitations, this study provides valuable insights into the prognostic interaction between clinical stage and structural remodelling in dogs with MMVD.

In conclusion, both the ACVIM stage and degree of LA remodelling were independently associated with survival in dogs with MMVD. These findings indicate that clinical decompensation remains the most powerful determinant of prognosis, but severe LA enlargement also identifies dogs at increased risk of mortality, even in the preclinical stage. Further prospective studies are warranted to validate these observations and to establish optimal monitoring and treatment strategies for dogs exhibiting advanced structural remodelling.
